# Functional connectome integration observed after antispastic epidural cervical spinal cord stimulation in a patient with TBI-induced disorder of consciousness: a case report

**DOI:** 10.3389/fnhum.2025.1533212

**Published:** 2025-06-20

**Authors:** Larisa Alexeevna Mayorova, Ekaterina Leonidovna Bondar, Margarita Leonidovna Radutnaya, Alexey Alexandrovich Yakovlev

**Affiliations:** ^1^Federal Research and Clinical Center of Intensive Care Medicine and Rehabilitology, Moscow, Russia; ^2^Institute of Higher Nervous Activity and Neurophysiology (RAS), Moscow, Russia

**Keywords:** disorders of consciousness, TBI, spinal cord stimulation, functional connectome, fMRI

## Abstract

We report the case of a patient with disease of consciousness who underwent 6 days of antispastic spinal cord stimulation followed by consolidation of a functional connectome as measured by resting state fMRI (rs-fMRI). The test spinal cord stimulation (SCS) system (with electrodes placed epidurally at the C3-C5 level) was used to evaluate its potential to relieve muscle contracture as the primary clinical target. Neurological and rs-fMRI examinations were performed before and after surgical placement of the spinal cord stimulation system. For neurological assessment of spasticity we used the Ashworth scale. To analyze fMRI, we used the extraction of functional connectivity coefficients and the construction of a connectivity matrixes. To construct a normative matrix of functional connectivity, 10 healthy volunteers of appropriate age were recruited as a control group. Analysis of rs-fMRI data showed that after a short course of epidural cervical spinal cord stimulation, the patient’s functional connectivity matrix similarity with the control group increased, which was manifested in the growth of ROI-to-ROI and inter-network functional connectivity coefficients. This finding may indicate the complexity of the neuromodulatory effect of spinal cord stimulation and its consolidating effect on the functional connectome of the brain, including brain regions associated with the function of maintaining arousal and awareness, even when the clinical effect is not perceptibly pronounced. We supposed to conduct the spinal cord therapy for this patient in a permanent way not only to relieve spasticity but also to support consciousness. We assume that functional connectome assessment in such clinical cases may help to give additional arguments in SCS-therapy prescription in patients with disorders of consciousness as well as to understand in the future the pathophysiological mechanism of the effect of this procedure.

## 1 Introduction

Spinal cord stimulation (SCS) is a therapeutic intervention that delivers electrical impulses to the spinal cord to manage a range of medical conditions. SCS is one of the most widely used invasive neurostimulation techniques. The procedure involves implanting one or more electrodes into the epidural space and delivering electrical current through the contacts. While SCS is primarily used in patients with disorders of consciousness (DoC) - a state where consciousness has been affected by damage to the brain - to manage pain and spasticity, some studies have reported improvements in the level of consciousness following cervical spinal cord stimulation (Della Pepa et al., 2013; Mattogno et al., 2017). Researchers suggest that this approach could be a promising treatment for DoC (Wang et al., 2020; Xu et al., 2019; Yang et al., 2022). However, since the initial reports, key questions remain unresolved: whether the observed effects result from direct modulation of neuronal signaling or improved hemodynamics (here, we focus solely on cervical SCS), the optimal stimulation parameters, and the overall efficacy in enhancing consciousness. These uncertainties stem partly from methodological challenges in such trials, including the lack of controlled conditions and difficulties in assessing treatment effects—particularly since clinical evaluation of consciousness in DoC patients is inherently complex. For instance, misdiagnosis rates of the vegetative state may reach 40% (Andrews et al., 1996; Childs et al., 1993; Schnakers et al., 2009; van Erp et al., 2015). A significant portion of these diagnostic errors stems from the phenomenon of latent consciousness (Owen et al., 2006) or cognitive-motor dissociation (CMD) (Bodien et al., 2024) in DoC patients. CMD is defined by the dissociation between a patient’s inability to execute observable motor commands and their preserved capacity for conscious brain activity modulation in response to instructions, as detected through neuroimaging (fMRI) or neurophysiological (EEG) methods. While the exact prevalence of CMD remains unclear, the largest multicenter study to date found that 25% of patients diagnosed with vegetative state or minimally conscious state-minus (based on CRS-R criteria) exhibited neural evidence of command-following (Bodien et al., 2024).

To address these limitations, we complemented clinical assessments with fMRI-based connectome analysis to objectively evaluate brain function. This report presents a unique case of cerebral connectome reorganization following a 6-day trial of anti-spastic cervical epidural SCS in a DoC patient.

## 2 Case presentation

A 29-year-old female was injured in a car accident in November 2019, was brought to the emergency room of the Emergency Department of the State Clinical Hospital in an extremely serious condition; CT scan of the brain verified an acute subdural hematoma in the area of the right frontal-parietal lobe with a maximum thickness of 13 mm. Resection decompressive skull trepanation in the right frontal-parieto-parietal region was performed, and acute subdural hematoma of right hemispheric localization was removed. She was admitted to the Federal Research and Clinical Center of Intensive Care Medicine and Rehabilitology in March 2020 in a serious condition to continue rehabilitation and treatment measures. Due to the increasing spasticity, it was recommended to perform antispastic epidural stimulation of the spinal cord.

The patient’s family history and personal medical history were unremarkable. Prior to the incident, the patient was essentially healthy.

### 2.1 Clinical examination and findings

Clinical examination at admission revealed a score of 8 on the Glasgow Coma Scale, and a score of 11 on the FOUR scale. The presence of an oral automatism reflex. Muscle strength could not be reliably determined due to limited contact with the patient. Passive movements in the extremities are limited due to increased muscle tone: flexor set of hands and feet. Muscle tone is elevated in the spastic type: in the right hand in the hand 4 points, proximally 3 points; in the left hand in the hand 4 points, proximally 2 points; in the right leg 2 points, in the left leg 3 points on the modified Ashworth spasticity scale. There are pathologic Babinski reflexes on 2 sides. No control of pelvic organ functions.

### 2.2 Neurological assessment before and after SCS

The patient underwent trial spinal cord stimulation from April 7 to 14, 2020. To monitor treatment effects, repeat clinical and instrumental examinations were performed. Neurological assessment and fMRI were conducted no more than 24 h before and after the stimulation period. Pre- and post-study neurological evaluation included an assessment of consciousness using the JFK Coma Recovery Scale-Revised (CRS-R) (Giacino et al., 2004) since CRS-R is established as the most reliable tool for chronic DOC assessment (Seel et al., 2010). Validation of the Russian adaptation of the Coma Recovery Scale-Revised was conducted in Research Center of Neurology (Moscow) from October 2016 until April 2017, registered at clinicaltrials.gov (Mochalova et al., 2018).

We assessed the level of spasticity using the Ashworth scale (AS). It provides a semiquantitative measure of the resistance to passive movement but has limited inter rater reliability. The AS is simple, requires no instrumentation and is easy and quick to carry out, and has been used in many studies (Bohannon and Smith, 1987). The CRS-R and the spasticity assessments were performed two times: on the day of pre- and postsurgical fMRI by two independent neurologists.

Neurological examinations were conducted by two independent neurologists from the Center, with no financial conflicts of interest. Both specialists received comprehensive training, including: (1) general intensive care unit protocols and (2) specific certification in administering the Russian-adapted version of the Coma Recovery Scale-Revised (CRS-R).

### 2.3 SCS-therapy: temporary electrode installation and stimulation protocol

According to the clinical manifestations the aim of achieving a certain cervical level was the objective that guided the installation of the electrodes. A trial epidural electrode was implanted in the C3 to C5 area under general anesthesia on April 7, 2020.

The choice of placement area was determined based on existing evidence that this leads location is reported in the literature to be effective for the treatment of spasticity of various etiologies (Waltz et al., 1987; Waltz et al., 1981) and, given its proximity to the central nervous system and the ascending activating reticular system in particular, is most commonly used in DoC patients to enhance consciousness (Xia et al., 2018).

**FIGURE 1:**
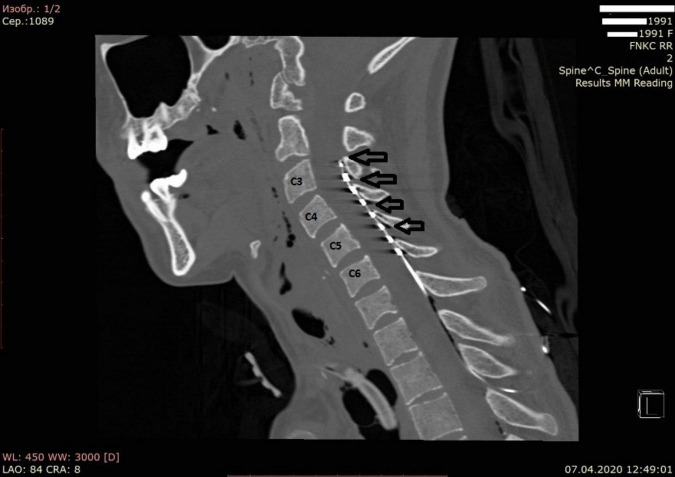
Computed tomography of the cervical spine in bone mode in the sagittal projection. The arrows indicate the position of the electrodes.

The patient was positioned on the left side and the head was secured using a Mayfield cranial stabilization system. The procedure included parasagittal transcutaneous puncture of the epidural space in the Th1-Th2 area with a Tuohy needle and insertion of the electrode in C3-C5 under intraoperative X-ray monitoring. An eight-pin monoaxial electrode (Octrode kit, St. Jude Medical, USA) was used. A test pulse generator (St. Jude Medical, USA: model 3599)^1^ was then connected to the distal part of the electrode to check impedance. The peripheral part of the electrode was fixed on the skin. The entire procedure took 15 min and was performed by a qualified neurosurgeon. A CT scan of the neck was performed after surgery to confirm the position of the electrode and rule out surgical complications (Figure 1).

The stimulator was connected on the 1st day after surgery. Stimulation options were isolated tonic mode, “Burst”, or combinations thereof. The decision to choose a mode was made based on the presence of clinical effect. With tonic mode the pulse width was set to 200 μs, and with “Burst” mode the pulse width was set to 1000 μs (5 pulses per burst). The stimulation frequency was 40 Hz for both modes. For testing the initial current was set at 1 mA, then it was gradually increased to the value at which the limb tremor appeared. Then the current was decreased by 1 mA. This was the value of the current at which the stimulation was performed. On April 14, 2020, following completion of six neuromodulation cycles, the trial stimulation period was concluded and the electrode array was explanted.

We used a cyclic stimulation mode of 15 min on and 5 min off in the daytime (from 8 a.m. to 8 p.m.) and turned the generator off at night to comply with the sleep– wake cycle. Contact 1 (the uppermost contact) or contact 2 of the electrodes were usually used as the stimulation contact. Stimulation parameters were selected according to previous data and the response of the patient. Stimulation contact and stimulation current were adjusted until the appearance of an expression of pain, consistent twitches of the extremities or significant alterations in respiration and heart rate, then, the stimulation current was turned down to an intensity at which no expression of pain, no consistent twitches of the extremities, and no significant alterations in respiration or heart rate occurred. Stimulation parameters were set at 2.5–3.5 mA with a frequency of 60 Hz and pulse width of 200 μs. Neuromodulation cycles were performed 4 times, with a break on weekends.

### 2.4 Functional MRI scanning parameters

Rs-fMRI examinations were performed before and after surgical placement of the spinal cord stimulation system. Magnetic resonance imaging (MRI) was performed on a Magnetom Essenza 1.5 T Scanner (Siemens, Germany). To acquire anatomical images in the sagittal plane, the T1 MPRAGE sequences were used with the following parameters: TR = 1900 ms, TE = 3.4 ms, 174 slices, slice thickness = 1 mm, FoV = 250 mm, reconstruction matrix of 256 × 256, voxel size = 1 × 1 × 1 mm. Resting-state fMRI was performed according to the following protocol: standard two-dimensional echo-planar imaging, TR = 3000 ms, delay = 0 ms, TE = 45 ms, 35 slices (ascending interleaved order), slice thickness = 5 mm, FoV = 192 mm, matrix of 64 × 64, voxel size = 3 × 3 × 3 mm, 180 measurements (volumes) for each subject.

### 2.5 Resting state data preprocessing pipeline and functional connectivity analysis

The fMRI data were processed on a PC HP Probook 430 G6 (no calculations were performed using a graphics processor) using MATLAB R2019b (MathWorks, Natick, MA, USA). Statistical Processing Package SPM12^2^ was used for preprocessing. The first two functional volumes were excluded from the analysis; the remaining images were aligned in time (for resting-state fMRI was not carried out) as well as relative to the first volume for movement artifacts correction. Further coregistration of the average functional image with the structural image was performed. The normalization procedure in the MNI space was performed using the DARTEL (Diffeomorphic Anatomical Registration Through Exponentiated Lie Algebra) tool and the New Segment Tool. Finally, the image was smoothed using a Gaussian filter with a core size of 8 × 8 × 8 mm.

After preprocessing, the CONN software application^3^ was used to create functional connectivity matrices from the patient’s fMRI data before the procedure, after the procedure, and for the average matrix of the normal reference group. The following steps are described for working with SPM12 preprocessed data to construct functional connectivity matrices. Denoising was performed by removing the following confounders by linear regression: the blood-oxygen-level dependent (BOLD) signal from the white matter and CSF masks (5 principal components of each signal); scrubbing (the number of regressors corresponded to the number of identified invalid scans identified using the Art toolbox^4^); [motion regression (12 regressors: 6 motion parameters, 16 first-order temporal derivatives)]. In addition, manually created lesion mask in the MRIcron toolbox (Rorden and Brett, 2000) was included in the denoising step to regress out any lesion-related signal. The resulting signals were band-pass filtered in the range of 0.008–0.12 Hz. Using the atlas built into the program, we analyzed the ROI-to-ROI functional connectivity of 165 brain areas and networks, including network interactions. The BOLD-signal time course of every ROI was calculated. The Fisher-transformed correlation coefficients between all pairs of these signals were computed, comprising a symmetric connectivity matrix. Three matrices were constructed: the patient’s preoperative and postoperative connectivity matrix and the average matrix of the reference group of healthy controls. The averaged matrix for the healthy control group (group characterization is given in paragraph 3.0) was constructed from individual matrices (from the concatenated 3-dimensional matrix) using MATLAB functions (see Supplementary materials, section a-b). Then all the obtained matrices were transformed into the graphical output of matrices using MATLAB function (see Supplementary materials, section c).

From the three acquired matrices, we derived additional matrices representing all brain areas (132 regions), cortical-cortical (91 regions) and subcortical interactions (15 regions) by selectively extracting specific subsets of data from the original matrices (see Supplementary materials, section d).

In the final data processing step, we computed the index of connectome intactness (ICI) for both pre- and post-intervention matrices, using the healthy control group as reference. ICI is a normalized measure comparing the similarity (or divergence) of an individual’s structural/functional connectome to a reference template derived from healthy controls. It reflects the degree to which network topology, edge weights, or nodal connectivity are preserved. As a similarity metric, we used the Pearson correlation coefficient. All steps for calculating ICI (matrix stretching and computing the Pearson correlation coefficient) were also performed in the MATLAB software environment using its standard functions (see Supplementary materials, section e). The description of the MATLAB code, including examples for each of the outlined steps, is provided sequentially in the Supplementary materials.

### 2.6 Spasticity before and after spinal cord stimulation

Overall, there was a slight decrease in spasticity according to both neurologists (Table 1). The level of consciousness according to the clinicians was unchanged, the patient understood simple instructions, but speech and purposeful behavior were absent. As a result of SCS therapy, some regression of pain syndrome was also recorded in the medical history. Neurological status without negative dynamics. The physicians assessing the patient before and after the procedure were blinded to her spinal cord stimulation treatment. While they were aware of a “spinal cord stimulation efficacy study,” they had no knowledge of whether she was in the active treatment or control group.

**TABLE 1:** Assessment of spasticity before and after 6 days of spinal cord stimulation.

Ashworth modified scale	Before SCS	After SCS	Δ
**Neurologist 1**			
Right hand	3	1	2
Left hand	3	1	2
Right leg	3	2	1
Left leg	3	2	1
**Neurologist 2**			
Right hand	3	2	1
Left hand	3	3	0
Right leg	1	2	−1
Left leg	3	3	0

### 2.7 Functional connectivity before and after spinal cord stimulation

**FIGURE 2:**
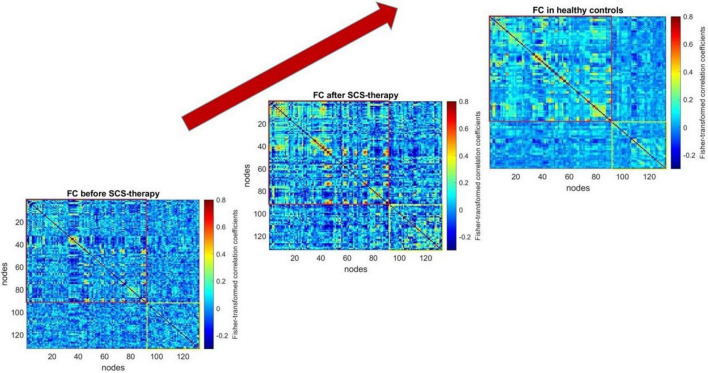
Functional connectivity matrices of the patient before and after 6 days of spinal cord stimulation and the healthy control group. The graphical representation of functional connectivity matrices, constructed using the CONN software application (http://www.nitrc.org/projects/conn), was generated with the MATLAB “imagesc” function. The top-right corner displays the averaged matrix (computed using MATLAB’s “mean” function) across the healthy control group. The script for constructing the figure can be found in the Supplementary Materials (section c).

Figure 2 shows from bottom to top the functional connectivity matrices of the patient before and after spinal cord stimulation and the healthy control group. The warmer shades reflect the higher degree of connectivity of the brain regions. Visually, the “after stimulation” matrix is more similar to the healthy control matrix than to the “before stimulation” matrix, which means that the patient’s total neuronal activity is closer to that of the healthy brain (both at the level of cortical-cortical interactions (red box) and subcortical interactions (yellow box).

To mathematically assess the degree of reorganization of the patient’s brain activity after 6-day spinal cord stimulation, we used the index of connectome intactness (Finn et al., 2015) calculated relative to the normal group. When assessing the changes in functional brain interactions in general after stimulation, some increase in the connectome integrity index was noted. Further, we made an attempt to evaluate in more detail which interactions contribute most to the obtained result and calculated the index separately for cortical-cortical and subcortical interactions. In all cases, we observed an increase in the connectome integrity index (relative to the control group) both at the level of cortical and subcortical interactions, with the greatest changes in the set of subcortical functional connections (Table 2).

**TABLE 2:** Changes in the connectome intactness index before and after 6 days of spinal cord stimulation.

Functional connectivity	ICI before SCS	ICI after SCS	Δ ICI
All brain areas (132)	0.41	0.44	0.03
Cortical-cortical interactions (91)	0.50	0.51	0.01
Subcortical interactions (15)	0.40	0.52	0.12

### 2.8 Follow-up and outcomes

**FIGURE 3:**
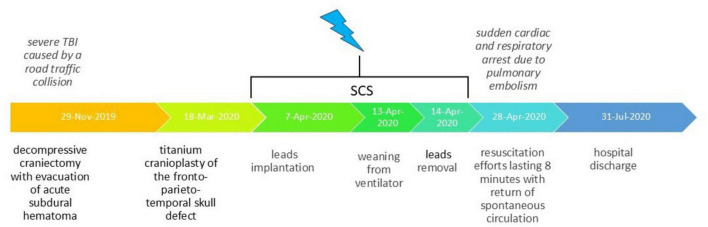
Historical and current information from this episode of care organized as a timeline.

On the penultimate day of spinal cord stimulation (13.04.2020), the patient underwent successful decannulation following clinical evaluation. She was subsequently transferred to the rehabilitation department for enhanced rehabilitation therapy. On April 28, 2020 (14 days after electrode removal), the patient experienced cardiac and respiratory arrest secondary to acute pulmonary embolism. The observed complication originated from deep venous system pathology in the lower limbs. After 8 min of resuscitation, spontaneous circulation was restored; however, the patient sustained severe anoxic brain damage. Given this confounding complication, subsequent evaluation of delayed spinal cord stimulation effects on consciousness was deemed clinically irrelevant and thus discontinued. The patient was discharged on July 31, 2020, in critical condition, remaining in a vegetative state with only minimal flexion in response to painful stimuli preserved. Thus, despite initially demonstrating highly favorable prognosis following the spinal cord stimulation course, the patient subsequently experienced cardiorespiratory arrest resulting in anoxic brain injury, which substantially worsened her clinical outlook. Historical and current clinical data for this care episode, organized as a timeline, are presented in Figure 3.

### 2.9 Healthy voluntaries

We scanned healthy volunteers using the same protocol to construct an average matrix of functional connectivity and further compare patient data with this mean functional connectivity matrix. The group comprised 10 healthy volunteers (5 women, median age 33 years, age range 25–41 years). The processing pipeline of the data completely coincided with those in the patients. Healthy participants of the control group were instructed to remain calm, with their eyes closed, not falling asleep and not thinking about anything. To minimize head movements, foam pads were used to fix the head during MRI acquisition.

## 3 Discussion

In this clinical case, despite demonstrating only a modest antispastic effect from cervical epidural spinal cord stimulation (SCS), we observed three notable neurofunctional changes: (1) improved normalization of the functional connectome metrics; (2) a distinct shift in subcortical functional connectivity patterns toward healthy control profiles; and (3) significant reorganization of functional connectivity architecture while behavioral consciousness levels remained unchanged, as assessed by clinical evaluation. Importantly, the observed enhancement in fMRI-derived functional connectome normalization metrics represents, to our knowledge, the first documented evidence of this phenomenon in such patients.

The observed increase in normalization metrics of functional connectivity matrices, particularly the Index of Thresholded Connectome Intactness (ITCI), correlates with higher levels of consciousness (Sinitsyn et al., 2018). Therefore, we cannot exclude potential positive effects on the patient’s consciousness level despite the absence of clinical manifestations. Notably, similar findings have been reported in EEG studies, which demonstrated increased complexity in cerebral cortical informational interactions following comparable interventions (Wang et al., 2020).

In this clinical case, the most pronounced changes in connectome intactness index were observed in subcortical regions. This finding partially aligns with existing literature: studies examining the effects of pain relieving SCS on brain function have reported bilateral enhancement of medial thalamic functional connectivity (Moens et al., 2012). However, other studies have primarily documented alterations in cortical functional connectivity, as evidenced by both EEG (Goudman et al., 2020) and fMRI data (De Ridder and Vanneste, 2016; Mayorova et al., 2023). It should be emphasized that this comparison between our anti-spastic therapy in a DoC patient and the cited studies on pain relieving or even anti-spastic therapy in conscious patients is, strictly speaking, methodologically limited. Within the context of our clinical case’s pathology, it would be more appropriate to reference studies investigating the unconscious connectome. Neuroimaging studies of DoC’s using fMRI have consistently demonstrated functional connectivity abnormalities at both cortical and subcortical levels. At the cortical level, these disruptions affect multiple resting-state networks (Athena et al., 2014; Demertzi et al., 2014; Wu et al., 2015; Yao et al., 2015), while subcortical impairments have been particularly documented in thalamic regions (He et al., 2015). Notably, the default mode network (Cauda et al., 2008; Fernández-Espejo et al., 2012) and overall connectome functional connectivity show progressive reduction in DoC patients that correlates with their consciousness level severity.

The appropriateness of selecting functional connectivity and connectome analysis based on fMRI data as an objective assessment method for interventions has been widely documented in numerous studies. Functional connectivity has emerged as a critical methodology for consciousness assessment (Höller et al., 2014; Sanders et al., 2012). Growing evidence suggests its particular utility in both diagnostic clarification of disorders of consciousness (DoC) and monitoring neurodynamic changes following cervical spinal cord test stimulation (Demertzi et al., 2014). Furthermore, contemporary research indicates that intra- and inter-network connectivity metrics may serve as valuable biomarkers for assessing functional brain impairment and predicting potential consciousness recovery (Di Perri et al., 2016; Silva et al., 2015).

The significant changes in the functional connectome obtained in this study against the background of clinically unchanged level of consciousness may be due to the imperfection of the available scoring methods. The clinical assessment of the level of consciousness, among other things, is also influenced by the preservation of the speech production function, about which we know nothing. There is no information in the medical history about the nature of the functional asymmetry of the patient’s brain before the disease. For these reasons at least, we could have missed, for example, a temporary increase in the patient’s level of consciousness. In addition, some authors believe that the process of pathological decrease in the level of consciousness (or emergence from unconsciousness) is progressive rather than discontinuous (i.e., not of the “all-or-nothing” type) in relation to, for example, chronic disorders of consciousness (Kotchoubey et al., 2014). Only in 2009 it was proposed to distinguish subgroups within the state of minimal consciousness according to the presence of signs of purposeful behavior of varying complexity; to date, there are only 3 such subgroups, and due to the limited behavior of patients for many other reasons, this number may not correspond to the number of patterns of brain organization within the state of minimal consciousness.

The current study has several important limitations that should be acknowledged. First, due to the inherent constraints of our study design, we cannot establish a definitive causal relationship between the observed functional connectome changes and the spinal cord stimulation procedure. However, the close temporal proximity of the observed effects to the intervention, combined with the characteristic progression patterns of this neurological condition, strongly suggests their potential association. Second, we were unable to evaluate the long-term effects of spinal cord stimulation because further follow-up became clinically unjustifiable after the patient developed pulmonary embolism and subsequent anoxic brain damage. Third, our analysis did not include assessment of cerebral white matter signals, which might have provided additional valuable information about network connectivity changes. Nevertheless, despite existing evidence supporting the informativeness of white matter signals (Schilling et al., 2023), we still lack definitive methods for interpreting these signals. The primary objective of our analysis was to construct functional connectivity matrices between gray matter regions and brain networks with relatively well-characterized functional distributions. Moreover, there is evidence suggesting white matter may contribute noise to the acquired signal. Considering combined factors - the challenges in white matter signal interpretation, our specific analytical goals, and potential artifacts - we concluded that excluding white matter data represents the most balanced approach. An additional justification for this decision lies in our scanner’s limited magnetic field strength (1.5T), which is suboptimal for obtaining informative signals from white matter and cerebrospinal fluid while risking contamination of the BOLD signal.

## 4 Conclusion

Our study, despite its shortcomings, is part of a body of accumulating evidence suggesting that SCS therapy may be effective in treating patients with DoC, not only in correcting spasticity and pain but also in improving brain function as an interconnected system. The data we obtained on the overall state of the functional connectome following a short cycle of SCS therapy appear promising for the clinical application of this method in DoC. We also demonstrated that the proposed method for assessing functional connectome changes can serve as a tool for rapid evaluation of the intervention’s efficacy and potential.

## Data Availability

The raw data supporting the conclusions of this article will be made available by the authors, without undue reservation.

## Footnotes

1
https://fda.report/GUDID/05415067016553

2
http://www.fil.ion.ucl.ac.uk/spm

3
http://www.nitrc.org/projects/conn

4
https://www.nitrc.org/projects/artifact_detect
