# Supplementary material for: Functional connectome integration observed after antispastic epidural cervical spinal cord stimulation in a patient with TBI-induced disorder of consciousness: a case report
Source: Front Hum Neurosci. 2025 Jun 20;19:1533212. doi: 10.3389/fnhum.2025.1533212 (PMC12226549; doi:10.3389/fnhum.2025.1533212)
Supplement: Supplementary file 1 [file Table_1.docx]

**The MATLAB code employed in the data processing pipeline**

(a) concatenation of z-scored functional connectivity matrices (ROI-to-ROI) across healthy controls (n=10):

< [C = cat(3,A1,A2,…,A10)](https://www.mathworks.com/help/matlab/ref/double.cat.html#f70-456598) >

(b) computing the mean of the concatenated three-dimensional matrix in healthy controls:

< M = mean(C, 3) >

(c) graphical representation of matrices in Figure 2, illustrated using the healthy control matrix
(functional connectivity matrices for the patient’s whole-brain data were plotted immediately after generation in the CONN toolbox, whereas the healthy control matrix was visualized after completing the steps of (a) concatenation and (b) averaging):

< imagesc(M(1:164,1:165)')

colorbar

h = colorbar;

ylabel(h, 'Fisher-transformed correlation coefficients')

title('FC in healthy controls')

xlabel('nodes')

ylabel('nodes')

ax=gca;

ax.FontSize=13;

set(gca,'xtick',0:20:165);

savefig('FC in healthy controls.fig'); >

(d) generation of additional matrices representing whole-brain (132 ROIs), cortico-cortical (91 ROIs), and subcortical (15 ROIs) connectivity through selective extraction of specific data subsets from the source matrices. The procedure was performed identically for each of the three original matrices; the healthy control matrix is presented here as an example:

< load('M.mat');

CortexSubHC=zeros(132);

CortexSubHC(1:132, 1:132)=M(1:132, 1:132);

save('CortexSubHC.mat', 'CortexSubHC');

CortexHC=zeros(91);

CortexHC(1:91,1:91)=M(1:91, 1:91);

save('CortexHC.mat', 'CortexHC');

SubcortexHC=zeros(15);

SubcortexHC(1:15,1:15)=M(92:106, 92:106);

save('SubcortexHC.mat', 'SubcortexHC'); >

(e) calculation of the Index of Connectome Intactness (ICI-index) for the patient's functional connectivity matrices before and after SCS therapy relative to the normative group (demonstrated using the whole-brain connectivity matrix comprising 132 connections):

1) vectorization of the upper triangular portion of symmetric matrices (performed identically for the patient's pre- and post-intervention connectivity matrices and the averaged control group connectivity matrix):

< load('CortexSubHC.mat');

n=132;

[ii,jj] = meshgrid(1:n,1:n);

stringedCortexSubHC= CortexSubHC(ii>jj);

save('stringedCortexSubHC.mat', 'stringedCortexSubHC'); >

2) calculation of the patient's Pearson linear correlation coefficient relative to healthy controls:

< [rho,pval] = corr(stringedCortexSubHC,stringedCortexSubPatientBefore, 'type', 'Pearson');

[rho,pval] = corr(stringedCortexSubHC,stringedCortexSubPatientAfter, 'type', 'Pearson'); >
